# Sensorineural Hearing Loss due to Air Bag Deployment

**DOI:** 10.1155/2012/203714

**Published:** 2012-06-12

**Authors:** Masafumi Ohki, Jyunichi Ishikawa, Atsushi Tahara

**Affiliations:** Department of Otolaryngology, Saitama Medical Center, Saitama Medical University, Saitama 350-8550, Japan

## Abstract

Deployment of the air bag in a passenger vehicle accident rarely causes otologic injuries. However, sensorineural hearing loss induced by air bag deployment is extremely rare, with only a few cases reported in the English literature. A 38-year-old man involved in a traffic accident while driving his car at 40 km/hour presented with right sensorineural hearing loss and tinnitus, without associated vertigo. Pure-tone audiometry demonstrated elevated thresholds of 30 dB and 25 dB at 4 kHz and 8 kHz, respectively, on the right side. Air bag deployment in car accidents is associated with the risk of development of sensorineural hearing loss.

## 1. Introduction

Passenger-vehicle air bags were invented for protection from various injuries in traffic accidents. Nowadays, vehicles usually come equipped with air bags. These air bags instantly inflate with a large volume of gas in the case of a car collision, creating a brief (<100 msec) and intense (150 to 170 dB) pressure wave [1]. Deployment of air bags has been reported to inflict damage on the face, neck, upper chest, and abdomen, for example, eyes, facial nerve, cervical spine, temporomandibular joint, upper airway, lungs, and heart [2–5]; however, air bag deployment has rarely been reported to cause otologic injuries. Sensorineural hearing loss due to air bag deployment is rare, and there have been only a few reports in the English literature [1, 4, 6–10]. Herein, we describe a patient with traumatic sensorineural hearing loss caused by air bag deployment.

## 2. Case Report

A 38-year-old man was involved in a traffic accident while driving his car at the speed of 40 km per hour. Instantly after the car collision, the air bag deployed in front of him. The patient was found to be conscious and to not have suffered from any damage of the brain. After the accident, the patient began to suffer from fullness and mild tinnitus in the right ear, without associated vertigo. His ear drums were normal, and a tympanogram showed an A-type result. Pure-tone audiometry demonstrated elevated thresholds of 30 dB and 25 dB at 4 kHz and 8 kHz, respectively, on the right side, suggestive of right sensorineural hearing loss. The patient had no history of hearing problems or tinnitus. He was treated by a single intravenous injection of 600 mg of hydrocortisone, followed by oral administration of prednisolone at the starting dose of 40 mg/day, over a 21-day tapering course. The hearing loss recovered partially to 15 dB and 15 dB at 4 kHz and 8 kHz, respectively. The right-sided tinnitus was relieved.

## 3. Discussion

**Figure 1:**
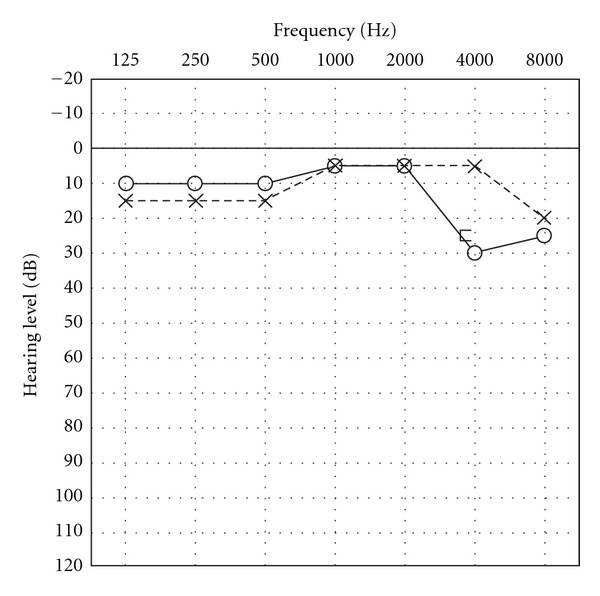
Audiograms show sensorineural hearing loss in the right ear.

Otologic injuries reported to be induced by air bag deployment include tympanic membrane perforation, conductive hearing loss, tinnitus, disequilibrium, and sensorineural hearing loss [9, 10]. Sensorineural hearing loss has been reported in the frequency range of mid- to high-tones in the majority. The most frequently affected frequency was 2 to 4 kHz [10]. Experiments in human volunteers [10] have demonstrated a temporary threshold shift after exposure to air bag deployment. Experiments in squirrel monkeys concluded no permanent sensorineural hearing loss at air bag deployment velocities up to 100 mph and SPL of 150 dB [11]. However, an experiment in cats showed immediate threshold shift to 60 dB, on average, at 4 kHz and a permanent shift to 37 dB, on average [12]. This report is consistent with our data and other previously reported data. However, hearing loss may not only be limited to the mid- to high-tone range, but also extended to other ranges of tone. The reported types of sensorineural hearing loss are varied, that is, dip-shaped, sloping, declining, and flat (Figure 1). The presumed mechanisms underlying the development of otologic injuries are acoustic trauma, rupture of the inner ear window due to mechanical deflection by a slap on the ear, and inner ear concussion syndrome. Sensorineural hearing loss is usually treated by steroid administration. However, the prognosis is not very good, and permanent sensorineural hearing loss often remains. We need to bear in mind the possibility of occurrence of sensorineural hearing loss due to air bag deployment in the case of car accidents.

## 4. Conclusion

We have presented a case of sensorineural hearing loss developing due to air bag deployment in a car accident. Pure-tone audiometry showed elevated thresholds at 4 and 8 kHz. Air bag deployment is associated with the risk of development of sensorineural hearing loss.
